# Optimization of small RNA library preparation protocol from human urinary exosomes

**DOI:** 10.1186/s12967-020-02298-9

**Published:** 2020-03-18

**Authors:** Dolores Olivares, Javier Perez-Hernandez, Daniel Perez-Gil, Felipe J. Chaves, Josep Redon, Raquel Cortes

**Affiliations:** 1 Cardiometabolic and Renal Risk Research Group, INCLIVA Biomedical Research Institute, Avd. Menéndez Pelayo, accesorio 4, 46010 Valencia, Spain; 2 Present Address: INSERM, U1016, Cochin Institute, 75014 Paris, France; 3 Present Address: Genomics England, Dawson Hall, Charterhouse Square, London, EC1M 6BQ UK; 4 Genomics and Diabetes Unit, INCLIVA Biomedical Research Institute, Avd. Menéndez Pelayo, accesorio 4, 46010 Valencia, Spain; 5 CIBER of Diabetes and Associated Metabolic Diseases (CIBERDEM), Institute of Health Carlos III, Minister of Health, Barcelona, Spain; 6 Internal Medicine, Clinic Universitary Hospital, Avd. Blasco Ibañez, 17, 46010 Valencia, Spain

**Keywords:** Exosome, microRNA, Urine, Adapter-dimer, Size selection step, Next generation sequencing

## Abstract

**Background:**

Sequencing of miRNAs isolated from exosomes has great potential to identify novel disease biomarkers, but exosomes have low amount of RNA, hindering adequate analysis and quantification. Here, we have assessed several steps in developing an optimized small RNA (sRNA) library preparation protocol for next-generation sequencing (NGS) miRNA analysis from urinary exosomes.

**Methods:**

A total of 24 urinary exosome samples from donors were included in this study. RNA was extracted by column-based methods. The quality of extracted RNA was assessed by spectrophotometric quantification and Bioanalyzer software analysis. All libraries were prepared using the CleanTag small RNA library preparation protocol and the effect of our additional modifications on adapter-dimer presence, sequencing data and tagged small RNA library population was also analyzed.

**Results:**

Our results show that good quality sequencing libraries can be prepared following our optimized small RNA library preparation protocol from urinary exosomes. When the size selection by gel purification step was included within the workflow, adapter-dimer was totally removed from cDNA libraries. Furthermore, the inclusion of this modification step within small RNA library protocol augmented the small RNA mapped reads, with an especially significant 37% increase in miRNA reads, and the gel purification step made no difference to the tagged miRNA population.

**Conclusions:**

This study provides researchers with an optimized small RNA library preparation workflow for next generation sequencing based exosome-associated miRNA analysis that yields a high amount of miRNA mapped reads without skewing the tagged miRNA population significantly.

## Background

Over recent years changes in circulating microRNAs (miRNAs) levels have been associated with a broad range of pathological processes. Their analysis offers various advantages which make them a potential goldmine in identification of novel biomarkers: (1) they can be found in non- or minimally invasive specimens; (2) they are relatively stable in clinical samples as regards RNase digestion, temperature variation and multiple freeze–thaw cycles; (3) they are involved in pathway regulation, showing tissue and cell-specific expression profiles [1]. Screening for miRNA signatures associated with different pathologies has an important role in clinical research [2–4]. The last few years have therefore seen increasing development and optimization of the various miRNA purification, detection and analysis protocols, of which next generation sequencing (NGS) is a powerful tool to detect RNA molecules in biological samples [5–7].

Our group has previously reported an association between exosomal miRNAs and albuminuria in hypertension, and renal damage in systemic lupus erythematosus [8, 9]. Exosomes, 40–130 nm membrane-derived vesicles, have been identified as novel carriers for intercellular genetic material exchange and communication [10, 11], containing various nucleic acid species including mRNAs, small non-coding RNAs (sncRNAs) and particularly miRNAs [12, 13]. Consequently, there is growing interest in their use as non-invasive biomarkers for disease diagnosis and for monitoring disease recurrence, overall comprehensive analysis of the entire miRNA repertoire of exosomes in important diseases such as cancer, immune disorders and cardiovascular disease [14–16].

However, there is a limiting factor, the lower amount of small RNA (sRNA) present in exosomes than in tissues, cell cultures or biofluids like plasma makes it difficult to obtain good quality sRNA libraries for NGS analysis. Thus, previous studies presented sRNA library protocols modified to low RNA template input to avoid adapter-dimer formation, for example with an extra adapter dilution, using chemical modified adapters or with an additional gel purification step [17, 18], but not with RNA from exosome samples. Accordingly, there is interest in developing an optimized protocol of small RNA libraries to avoid this problem.

The objective of this study is to provide researchers with an improved sRNA library preparation workflow for NGS analysis of miRNAs from urinary exosomes. We tested the effect of low RNA input and an additional purification step on adapter-dimer formation, sRNA mapped reads and tagged library population.

## Methodology

### Samples

A total of 24 samples of urinary exosomes from donor patients were analyzed and raw data are included in the BioProject PRJNA590749, (Additional file 1: Table S1). All samples were processed in duplicate, one without size exclusion of PCR products and other using Polyacrylamide Gel Electrophoresis (PAGE) gel purification for size exclusion of PCR products. Fresh, first morning urine samples (50 mL) were collected in sterile containers and processed within 1 h after collection. Written informed consent was obtained from all donors and the study was approved by the Ethics Committee of the Hospital Clínico Universitario of Valencia and performed according to the Declaration of Helsinki.

### Isolation of urinary exosomes

Exosomes were isolated from urine specimens using a combination of centrifugation, ultracentrifugation, and DTT treatment, as previously described [8]. In brief, urinary cells and debris were removed by centrifugation at 2250*g* for 30 min at 4 °C. Next, 50 mL of the collected supernatant were transferred to clean tubes with 4.2 mL of protease inhibitor cocktail (Sigma, Missouri, USA) and centrifuged at 20,000*g* for 45 min at 4 °C to eliminate large microvesicles (Ultracentrifuge Optima L 100 K, 70 Ti rotor, Beckman Instruments, CA, USA). The supernatant was spun in an ultracentrifuge at 121,000*g* for 70 min at 4 °C, obtaining exosome-depleted supernatant. Exosome pellets were treated with DTT to eliminate protein complexes, washed with sterile RNase-free PBS and ultracentrifuged again at 121,000*g* for 70 min (Ultracentrifuge Optima L 100 K, 70.1 Ti rotor, Beckman Instruments, CA, USA). Exosome pellets from 50 mL urine were suspended in 100 μL of sterile RNase-free PBS and immediately processed to extract RNA, as described below.

### RNA extraction

Total RNA was extracted from exosome pellets in 100 μL of exosome suspension using a Total exosome RNA and protein isolation kit (Invitrogen, Life Technologies, CA, USA) according to the manufacturer’s instructions, and stored at − 80 °C. Total RNA was quantified with NanoDrop ND-1000 spectrophotometer (Thermo Fisher Scientific, Waltham, MA, USA), and 2100 Bioanalyzer (Agilent^®^ Technologies, Inc., Santa Clara, CA, USA). A RNA 6000 Pico chip run was performed afterwards for analysis and quantification of RNA eluates. The extracted RNA was stored at − 80 °C until further analysis.

### Small RNA sequencing

sRNA transcripts were converted into barcoded cDNA libraries. Library preparation was performed with CleanTag Small RNA library preparation (TriLink Biotechnologies, San Diego, USA) followed by sRNA-Seq on the Illumina HiSeq 2000 platform (CNAG, Barcelona, Spain). This sRNA library kit contains chemically modified adapters and reagents to convert sRNA to corresponding cDNA libraries for NGS, suppressing adapter-dimer formation, which is optimized for low total RNA template input [17]. Limited RNA quantity from urinary exosome specimens led to library preparation following 10 ng total RNA template input. Multiplex adaptor ligations, reverse transcription primer hybridization, reverse transcription reaction and the PCR amplification were processed following library preparation protocol (Protocol # L-3206, TriLink Biotechnologies, San Diego, USA) according to the manufacturer’s instructions. When working with lower RNA input, the protocol offers modifications at several steps, for example a 1:4 adapter dilution in the adapter ligation step and 18 cycles for PCR amplification. These modifications, together with the use of chemical CleanTag modified adapters, are designed to improve ligation efficiency and eliminate adapter-dimer formation. We used the Index Primer Set 1 (Primers 1–12 with RT) and Index Primer Set 2 (Primers 13–24) from Illumina^®^ (Illumina, San Diego, CA, USA).

After PCR pre-amplification, the cDNA constructs were loaded onto the ABI 3730 (Applied Biosystems, CA, USA) for DNA fragment analysis by capillary electrophoresis according to manufacturer’s protocol. This method measures the relative size of DNA fragments with very high resolution and reproducibility, by capillary electrophoresis of fluorescent labelled DNA fragments, using internal fluorescent size standards (GeneScan™ 500 LIZ™ dye Size Standard, ThermoFisher, Massachusetts, USA).

Electropherograms obtained were analyzed with GeneMapper software 5.0 (Applied Biosystems, CA, USA). The size selection of amplified cDNA libraries are described in the section below. The cDNA libraries were qPCR-quantified using a KAPA library quantification kit by LightCycler 480 II (Roche, Basilea, Germany). A final concentration of 20 nM per library was used to generate the two pools, with or without the size selection step (24 samples per pool). Experiments were designed containing 24 samples which would be barcoded, pooled equally, and then loaded onto one lane of a flow cell. Libraries were prepared individually and barcoded with reverse primers during the PCR step which contained Illumina-compatible indices #1–24. The sequence libraries obtained were subjected to the Illumina sequencing pipeline, passing through clonal cluster generation on a single-read flow cell by bridge amplification on the cBot (TruSeq SR Cluster Kit v3-cBOT-HS, Illumina, San Diego, CA, USA) and 50 cycles were sequenced by synthesis on the HiSeq 2000 (Illumina, San Diego, CA, USA).

### Size selection of amplified cDNA libraries

**Fig. 1:**
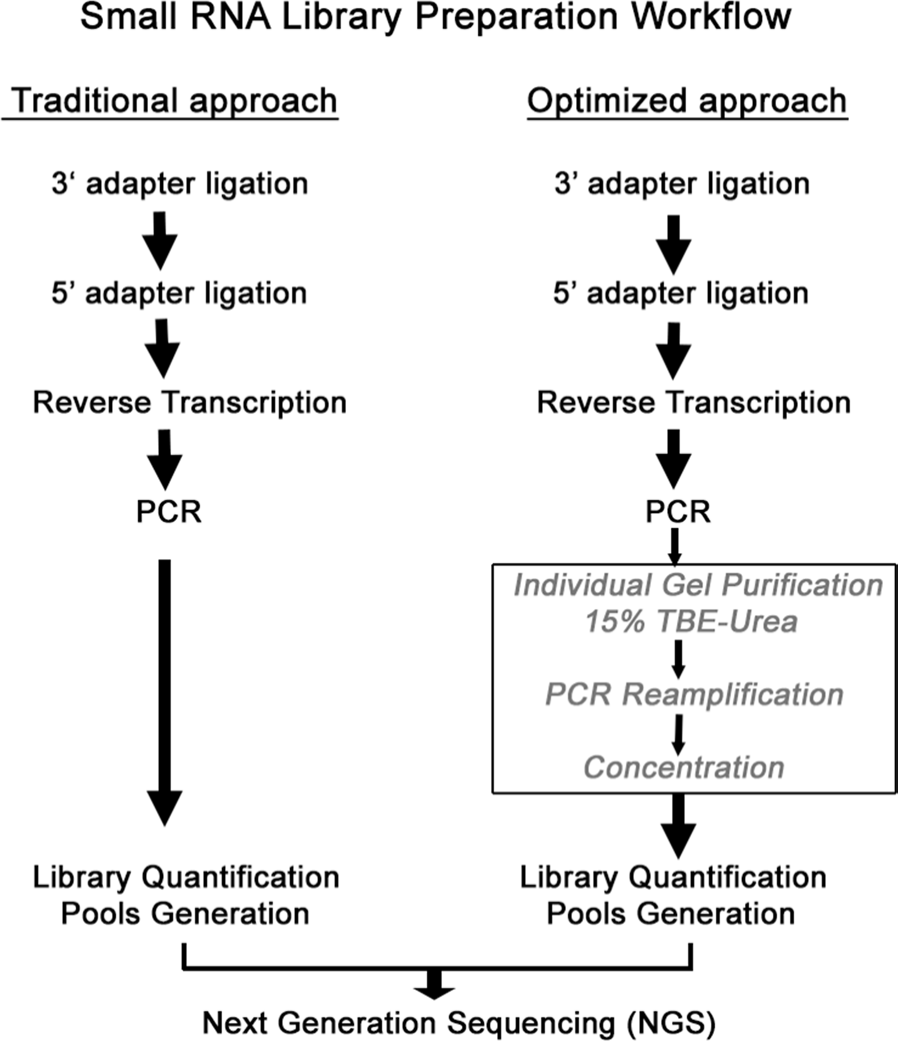
A brief work-flow chart for optimized Small RNA Library Preparation. Comparison between the traditional method and our optimized Small RNA Library Preparation method from urinary exosomes, indicating the new additional steps (inside square)

To test whether size selection was a necessary step to obtain higher quality sequencing data, 5 µL of PCR products together with 5 µL TBE-Urea Sample Buffer were loaded onto TBE-Urea gel (15%) in a vertical electrophoresis chamber, XCell Sure Lock Mini Cell (Invitrogen, Carlsbad, CA, United States), at 180 V and 13 mA for 4 h at room temperature. Next, gels were stained at 1× dilution for 20 min at dark (GelStar nucleic acid gel stain, Lonza, Basilea, Switzerland) and washed three times afterwards. To ensure the correct size of extracted bands, we developed and included in the gel three amplicons with different known sizes as markers (120 pb, 140 pb and 150 pb) corresponding to adapter-dimer, miRNAs and other sRNA species, respectively. Bands of samples, approximately 138 bp to 152 bp in size were cut out and passed on to gel extraction with 100 µL of Tris–EDTA in individual eppendorfs, then stored for 30 min at − 80 °C until frozen solid. Next, they were quickly thawed for 5 min at 95 °C in a thermal block, to ensure optimal recovery to PCR products. This freeze-rapid thaw approach greatly increases yield by allowing ice crystals to break apart the acrylamide matrix. Homogenates were centrifuged at 13,000 rpm for 2 min at room temperature to remove gel debris and collect supernatants containing clean libraries. Afterwards, the libraries were re-amplified in 10 cycles of PCR amplification with QIAGEN Multiplex PCR Kit (Qiagen, Hilden, Germany), obtaining a volume of 60 µL for each library. Finally, the cDNA constructs were purified and concentrated to 25 µL final volume with the QIAquick PCR Purification Kit (Qiagen, Hilden, Germany). A brief work-flow chart figure to side by side compare the tradition method and the optimized Small RNA Library Preparation method was included (Fig. 1).

### Software, statistics and mapping

To assess overall NGS data quality, adaptor sequences were trimmed from the 3′ end using Cutadapt (v.1.2.1), and reads without detectable adaptors were excluded from the data set. Sequence length distribution and base call accuracy were calculated with FastQC high throughput sequence data quality control software (Babraham Bioinformatics, UK, Version 0.11.4). This checking was performed before and after read cleaning. Trimmed reads were selected within desired ranges (18–26 bp and 24–33 bp) and aligned with Bowtie 2 v.0.12.7 [19], against the most recent human reference genome (GRCh38). Next, aligned reads were analyzed using HTSeq [20] against different databases to count how many reads mapped to each feature. In this study, miRNA features were obtained from the miRBase Sequence Database (Release 21) [21], piwiRNA features from the piRNA Bank (2018-12-09) [22], and other non-coding RNA features from NONCODE (2018) [23]. Statistical analyses were completed using GraphPad Prism software (GraphPad Software, Inc. La Jolla, CA, USA). Paired two-tailed Student’s T test at significance level of p < 0.05 was used to compare the effect of size selection on the small RNA mapped reads and miRNA sequencing, between purified and non-purified samples.

## Results

### Modification of small RNA protocol does not suppress adapter-dimer formation

**Fig. 2:**
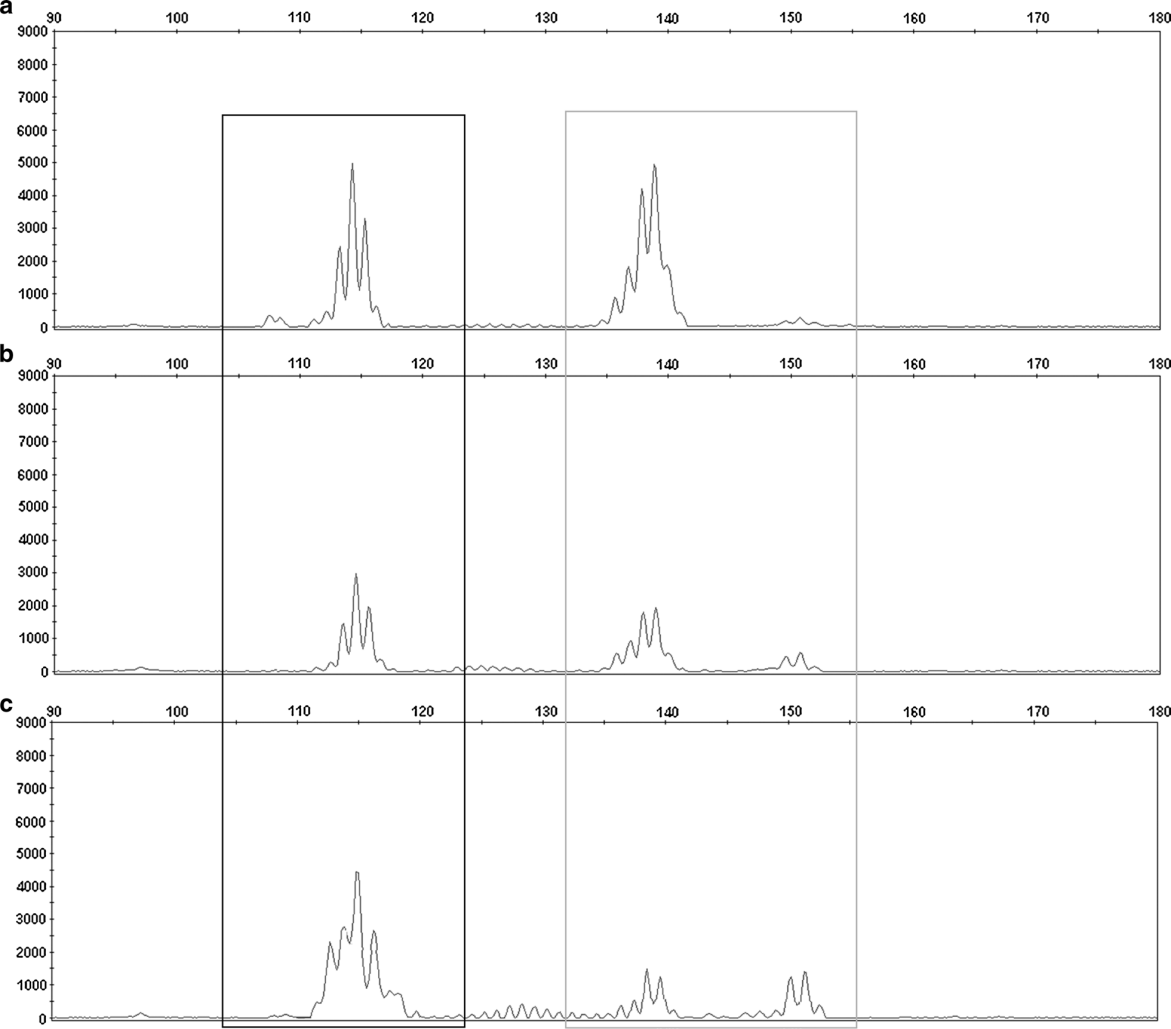
Electropherograms obtained for DNA fragment analysis by capillary electrophoresis to analyze adapter-dimer formation. Three different cDNA libraries from urinary exosomal samples showed the presence of adapter-dimer (black box), high amount of adapter-dimer with high cDNA library (grey box) (**a**), low amount of adapter-dimer with low cDNA library (**b**), and high amount of adapter-dimer with low cDNA library (**c**)

The small RNA library prep kit allowed us to obtain the corresponding libraries for next-generation sequencing from urinary exosomes. However, the modified adapters used did not prevent adapter-dimer formation from exosome samples (Fig. 2). Electropherograms obtained for DNA Fragment Analysis by Capillary Electrophoresis showed a peak at 110–120 bp that corresponds to the amount of adapter-dimer (black box) and other peaks at 135–155 bp that correspond to small RNA types, miRNA and other sRNA (grey box). The high peak of sRNA libraries was accompanied by high levels of adapter-primer (Fig. 2a), and a low amount of sRNA was also accompanied by low or high adapter-dimer formation (Fig. 2b, c, respectively).

**Fig. 3:**
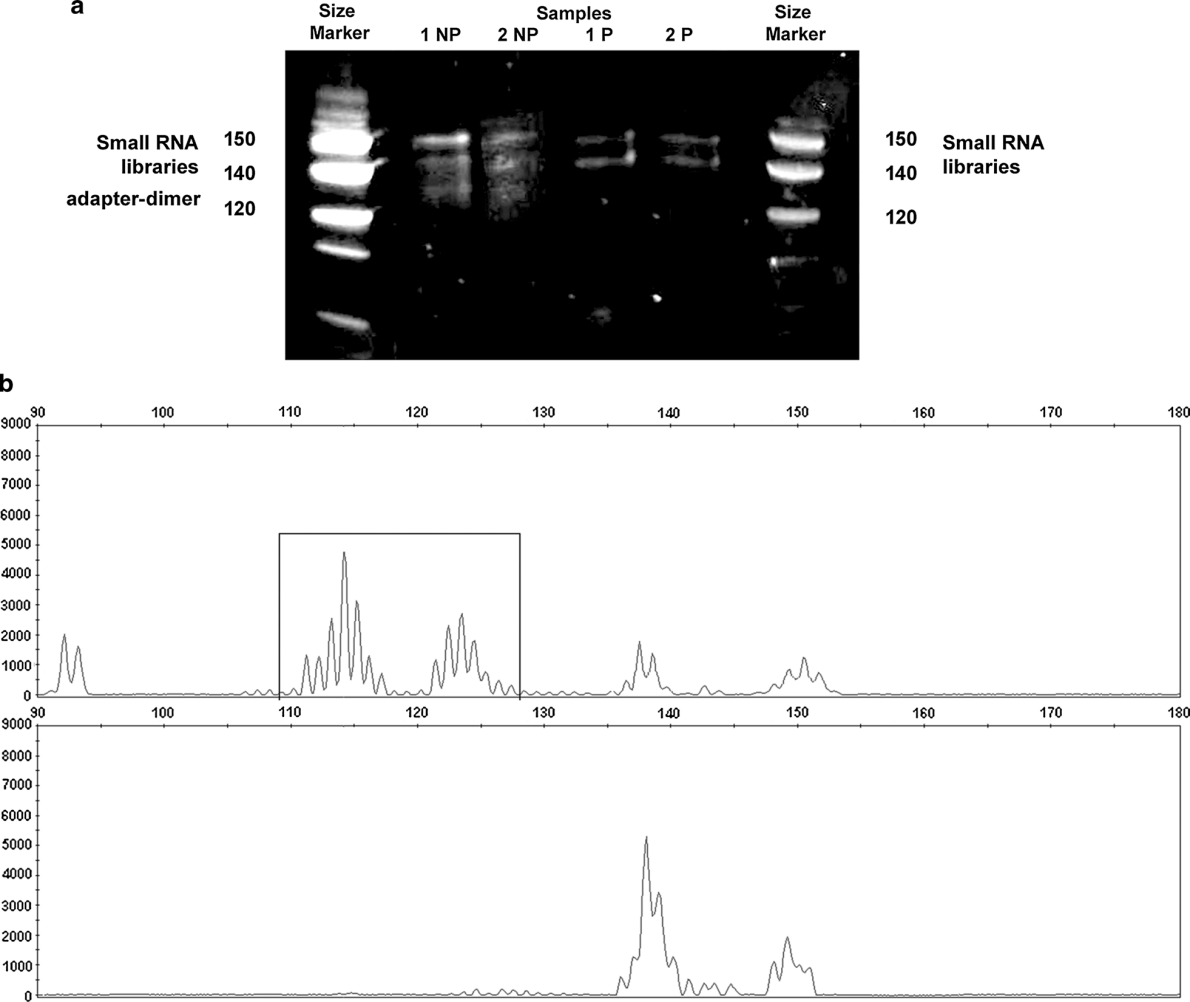
Comparison of adapter-dimer formation in small RNA libraries including size selection step by gel within the workflow. **a** Gel TBE-Urea staining showed the presence of adapter-dimer (around 120 nt in size) in non-purified (NP) samples, and it was removed in purified (P) samples 1 and 2. **b** Electropherogram showed elimination of adapter-dimer (black box) from the purified exosomal cDNA library, and an increase in the small RNA library amount

After CleanTag protocol optimization with an additional size selection step, we observed adapter-dimer formation in the gel before band extraction in non-purified (NP) samples 1 and 2, but no presence in purified (P) samples 1 and 2 (Fig. 3a). As electropherograms showed, adapter-dimer (black box) was totally removed after gel purification, and the sRNA library was enriched in the exosomal sRNA reads (Fig. 3b).

### Comparison of sequencing data including the size selection by gel within the CleanTag workflow

**Fig. 4:**
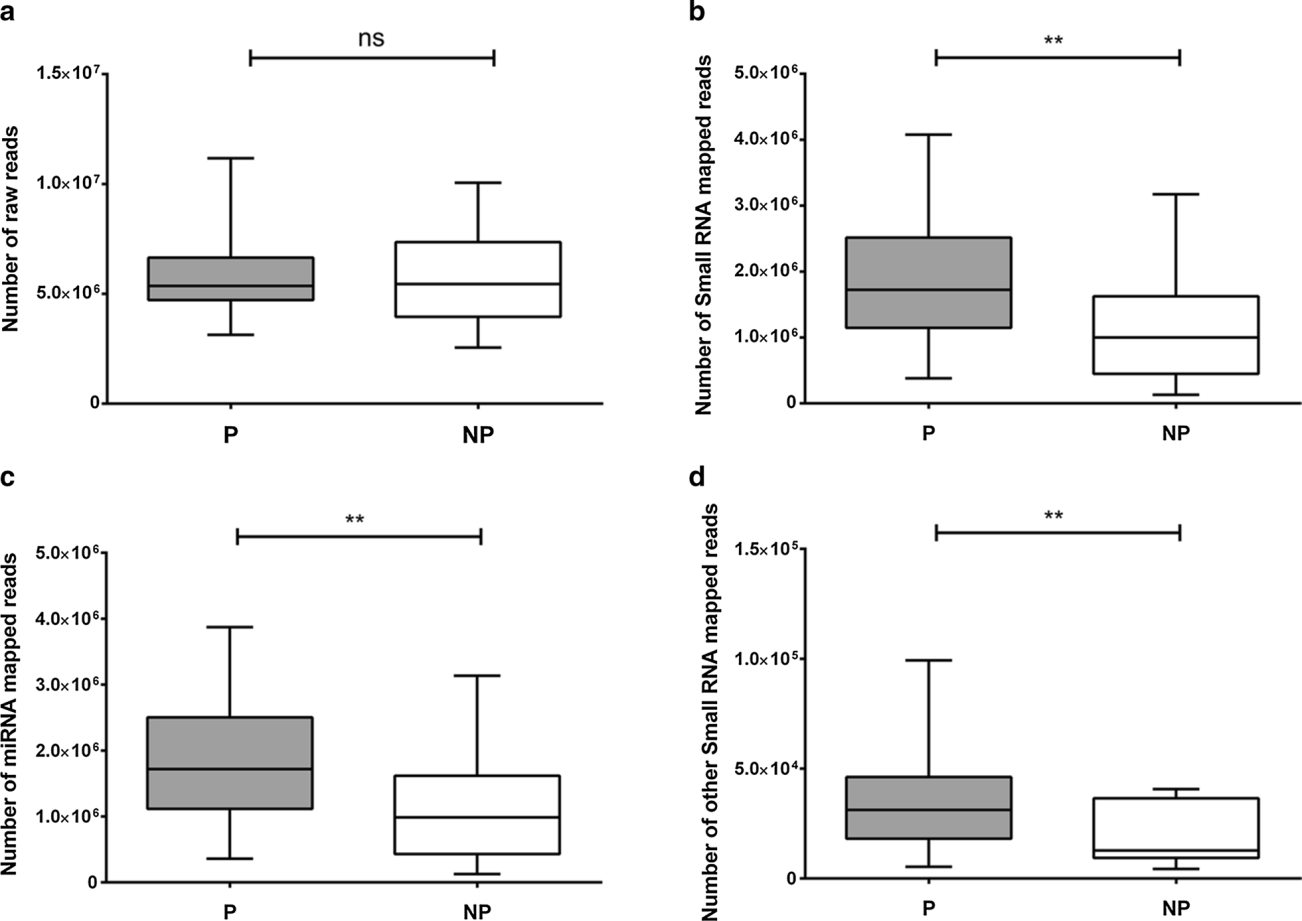
Comparison of sequencing data including size selection step by gel within the workflow. Sequencing reads obtained from 24 urinary exosomal samples in each condition, purified (P) and non-purified (NP) condition. Analysis of total raw reads (**a**), small RNA mapped reads (**b**), miRNA mapped reads (**c**) and other small RNA mapped reads (**d**) between groups. Statistical significance: **p < 0.01

When analyzing these two workflows with and without using a gel purification step, the total raw reads were statistically comparable, with slightly increase in non-purified samples (1.3%) (Fig. 4a). However, sRNA mapped reads were higher in gel-purified samples with a 41% increase compared to non-purified ones (p = 0.007) (Fig. 4b), representing 33% of total raw reads compared to the 20% in non-purified samples (p = 0.009).

In addition, when we compared the effect of gel purification on miRNA mapped reads alone, we found that purified samples had a significant 37% increase compared to non-purified ones (p = 0.008), and they represented 33% of the total raw reads compared to 19% of non-purified ones (p = 0.011) (Fig. 4c). From the other sRNA group analyzed, we obtained a two-fold increase in mapped reads between purified and non-purified samples (p = 0.027) (Fig. 4d). There was enrichment in sRNA reads, overall in miRNA data.

### Effect of size selection of PCR products by gel purification on tagged small RNA library population

**Fig. 5:**
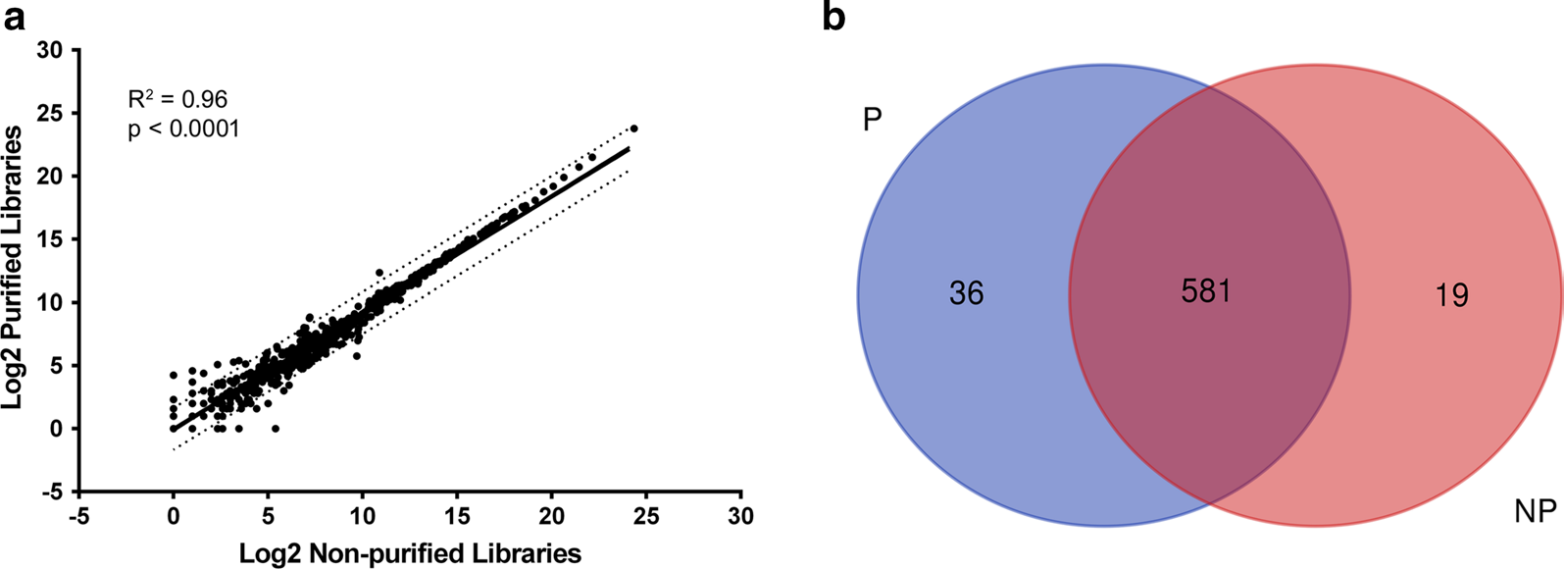
Effect of size selection step on miRNA tagged library population. **a** Correlation plot of purified and non-purified libraries using the CleanTag library prep. Tagged miRNA are plotted after log^2^ transformation. **b** Venn diagram of purified (P) and non-purified (NP) libraries depicting number of urinary exosomal miRNA identified in all 24 samples for each condition

In order to determine whether the size selection step produced any meaningful changes in miRNA detection, we compared sequencing results with or without gel purification within our optimized workflow. We found a similar tagged miRNA population with or without the size selection step, obtaining a determinant coefficient of R^2^ = 0.96, p < 0.0001 after log^2^ transformation (Fig. 5a). Furthermore, comparison of the two conditions with a Venn diagram showed that the purification protocol tagged more specific miRNA than the one without purification, although the majority (581 miRNA) of the tagged miRNA population were similar in the two conditions (Fig. 5b).

**Fig. 6:**
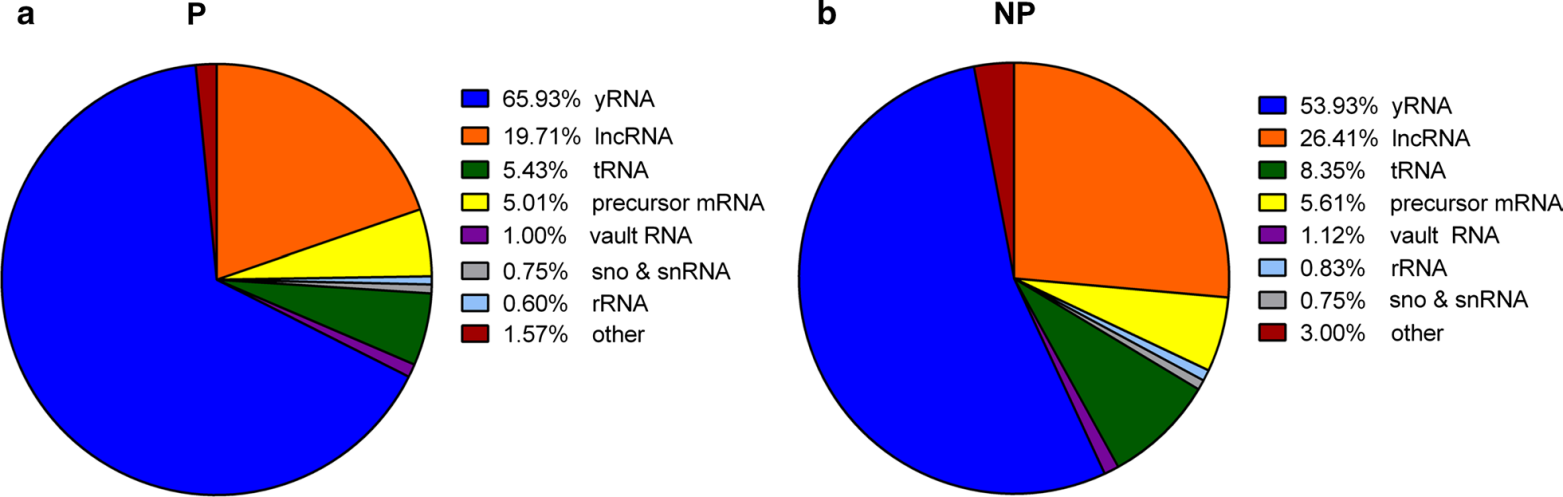
Effect of size selection step on the other ncRNA reads mapped. Pie chart showing the percentage of the other non-coding RNA (ncRNA) reads mapped in non-purified (**a**) and purified libraries (**b**). *lncRNA* long non coding RNA, *rRNA* ribosomal RNA, *tRNA* transfer RNA, *snoRNA* small nucleolar RNAs, *snRNA* small nuclear RNA

Finally, we analyzed the effect of gel purification on the other non-coding RNA (ncRNA) types. As with the miRNA population, the other ncRNA did not change in distribution, obtaining similar percentage between ncRNA types (Fig. 6a, b). In both purified and non-purified conditions, Y-RNA were the most representative (66% and 54%, respectively), followed by lncRNA (20% and 26%, respectively) and tRNA (5% and 8%, respectively).

## Discussion

This study presents an optimized Small RNA library preparation workflow for NGS-based exosome-associated miRNA analysis. We completely eliminated adapter-dimer formation and the sRNA mapped read count was increased, overall for miRNAs, without changing tagged miRNA population.

The low quantity of RNA present in exosomes is a limiting factor for researchers. In response to this, a variety of small RNA sample preparation kits have implemented several modifications in their workflows to avoid adapter-dimer formation and increase sRNA enrichment in samples with low RNA template input. As an example, to optimize sequencing performance the CleanTag Small RNA library prep kit builds libraries using specific adaptors designed for small amounts of starting material, suppressing adapter-dimer formation and eliminating the need for gel purification [17]. TruSeq^®^ Small RNA Sample Prep Kit and NEBNext Multiplex Small RNA Library Prep Set always implemented PAGE gel size-selection [24, 25]. Many recent clinical studies have utilized these kits to analyze differential miRNA expression in exosome samples [26, 27], hence the vital importance of analyzing the potential effect of these modifications on miRNA data obtained by NGS from urinary exosomes.

Our results showed that libraries from urinary exosomes could be prepared using CleanTag Small RNA library prep, although it failed to prevent adapter-dimer formation entirely. We obtained high cDNA libraries with a high amount of adapter dimer and low cDNA libraries with high or similar adapter-dimer. This fact indicates the need for a size selection step to purify the libraries for sRNA sequencing. When we analyzed the effect of gel purification on sRNA reads, we were able to demonstrate that including size selection within workflow efficiently tags sRNA and produces significantly more valuable reads and adapter dimer reduction compared to the protocol without gel purification. The robustness of our study lies in the analysis of results obtained from 24 individual cDNA libraries from urinary exosomes, which are more specific and sensitive for testing changes than working with library pools.

Human exosomes samples contain low abundance of sRNAs, and a unique or enriched set of miRNA compared to other EV subpopulations and cells [28, 29]. Identifying an exosomal miRNA signature associated with a pathological cellular state or disease has become increasingly important in recent years [15, 16]. In this regard, to develop a protocol for enrich miRNA mapped reads from exosome samples. Our improved protocol showed not only an increase in miRNA mapped reads, but also no changes in the tagged miRNA population. Though there are slight differences between the two conditions, overall among miRNAs that had very low reads, these results provide evidence that within our workflow the size selection step itself produces an unbiased tagged miRNA population. Furthermore, the number of mapped reads was augmented in the other sRNA species, without changes in the profile.

These findings prove that gel purification is specific for isolating the miRNA population from the urinary exosomes. This is because we were able to manually and carefully cut the band to between 145 and 160 nt, corresponding to miRNAs from the gel and avoid any other smaller or larger RNA populations in the samples. Further studies that analyzed exosome miRNA from other tissues it would be more universal. However, we think that whether this optimized protocol yields a higher amount of miRNA mapped reads without affecting the tagged miRNA population in urinary samples (biofluid with lower RNA input than plasma or plasma exosomes), could be applicable across other biofluids. We found purification by PAGE gel to be a very precise and specific method, particularly suitable for small sample size projects where miRNAs are the main focus.

## Conclusion

Our results present an optimized NGS-based library preparation workflow for exosome-associated miRNA analysis. Firstly, adapter-dimer formation was totally eliminated from urinary exosome cDNA libraries. Secondly, this study provides evidence that within our small RNA library preparation workflow, the size selection step itself obtained high miRNA mapped reads from urinary exosomes without skewing the tagged miRNA population. Therefore, in order to prepare sRNA libraries for NGS-analysis from samples with low RNA input, it is important to carefully analyze the effect of their adapted library preparation workflow, to further validate the NGS-data obtained.

## Supplementary information

**Additional file 1: Table S1.** Sequence Read Archive (SRA) and BioSample accession IDs of the samples analyzed.

## Data Availability

All data analysed during this study are included in this published article. The raw data are included in the BioProject PRJNA590749 and the corresponding Sequence Read Archive (SRA) and BioSample accession IDs are available in Additional file 1: Table S1.

## Abbreviations

cDNA: Complementary DNA
miRNA: microRNA
ncRNA: Non-coding RNA
NGS: Next generation sequencing
PAGE: Polyacrylamide Gel Electrophoresis
sRNA: Small RNA
TBE: Tris–Borate–EDTA
